# TGF-*β* and TGF-*β*/Smad Signaling in the Interactions between *Echinococcus multilocularis* and Its Hosts

**DOI:** 10.1371/journal.pone.0055379

**Published:** 2013-02-06

**Authors:** Junhua Wang, Chuanshan Zhang, Xufa Wei, Oleg Blagosklonov, Guodong Lv, Xiaomei Lu, Georges Mantion, Dominique A. Vuitton, Hao Wen, Renyong Lin

**Affiliations:** 1 State Key Lab Incubation Base of Xinjiang Major Diseases Research (2010DS890294) and Xinjiang Key Laboratory of Echinococcosis, First Affiliated Hospital of Xinjiang Medical University, Urumqi, Xinjiang, China; 2 Department of Nuclear Medicine, University of Franche-Comté and Jean Minjoz University Hospital, Besançon, Franche-Comté, France; 3 WHO-Collaborating Centre for the Prevention and Treatment of Human Echinococcosis, University of Franche-Comté and University Hospital, Besançon, Franche-Comté, France; Centro di Riferimento Oncologico, IRCCS National Cancer Institute, Italy

## Abstract

Alveolar echinococcosis (AE) is characterized by the development of irreversible fibrosis and of immune tolerance towards *Echinococcus multilocularis (E. multilocularis)*. Very little is known on the presence of transforming growth factor-*β* (TGF-*β*) and other components of TGF-*β*/Smad pathway in the liver, and on their possible influence on fibrosis, over the various stages of infection. Using Western Blot, qRT-PCR and immunohistochemistry, we measured the levels of TGF-*β*1, TGF-*β* receptors, and down-stream Smads activation, as well as fibrosis marker expression in both a murine AE model from day 2 to 360 post-infection (p.i.) and in AE patients. TGF-*β*1, its receptors, and down-stream Smads were markedly expressed in the periparasitic infiltrate and also in the hepatocytes, close to and distant from AE lesions. Fibrosis was significant at 180 days p.i. in the periparasitic infiltrate and was also present in the liver parenchyma, even distant from the lesions. Over the time course after infection TGF-*β*1 expression was correlated with CD4/CD8 T-cell ratio long described as a hallmark of AE severity. The time course of the various actors of the TGF-*β*/Smad system in the *in vivo* mouse model as well as down-regulation of Smad7 in liver areas close to the lesions in human cases highly suggest that TGF-*β* plays an important role in AE both in immune tolerance against the parasite and in liver fibrosis.

## Introduction

Alveolar echinococcosis (AE) is a rare, but severe zoonotic helminthic disease due to the proliferation of the larval stage of cestode *Echinococcus multilocularis (E. multilocularis)* [1]. In humans, accidental intermediate hosts, the severity of this disease results from both a continuous asexual proliferation of the metacestode and an intense inflammatory granulomatous infiltration around the parasite which causes pathological damages in the liver. The lesions act like a slow-growing liver cancer, progressively invading the neighboring tissues and organs. Granulomas around the parasitic vesicles, extensive fibrosis, and necrosis are the characteristic pathological findings [2]. Studies performed in the 1980s–1990s showed that dense and irreversible fibrosis composed of thick concentric bundles of heavily cross-linked type I and type III collagens surrounded the parasitic vesicles, and that *α*-smooth muscle actin (*α*-SMA)-expressing myofibroblasts (MFB) derived from the hepatic stellate cells (HSC) could play an important role in fibrosis development [3]–[7]. The diffusion of the fibrotic process even far from the parasitic lesions strongly suggested that cytokines produced in the periparasitic area could be involved in collagen synthesis, locally in the lesions and also in the liver distant from the lesions; it was also suggested that cytokines might be involved in the cross-linking of the collagen bundles. Little evidence, however, has been given until now on how *E. multilocularis* metacestode interacts with its host to promote fibrosis and especially on the nature and role of cytokines in fibrosis development in AE.

**Figure 1:**
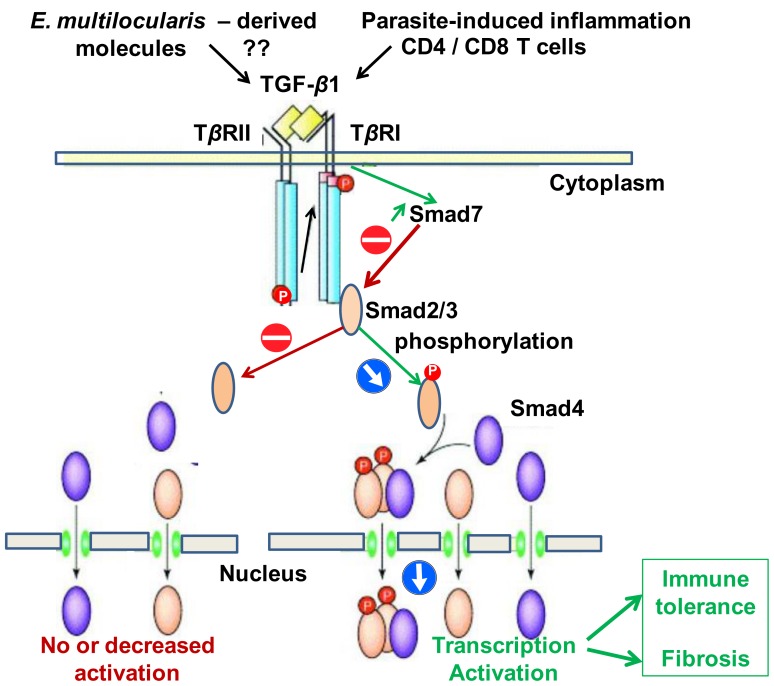
The TGF-*β*/Smad pathway; hypothesis for its involvement in the host-parasite relationship in *E. multilocularis* infection.

TGF-*β* is a major regulator of the immune responses, inducing and maintaining T-regulatory cells, reducing cytotoxic effector immune response and balancing the tolerogenic and immunogenic forces at play in various physiological states and chronic diseases, such as fetus growth and survival during gestation [8], cancer [9], chronic inflammatory diseases [10], or chronic and allergic respiratory diseases [11]. In these conditions, this polypeptide also regulates a variety of cell events involved in tissue regeneration and fibrosis. Similarly, its role has been recognized both to induce and maintain immune tolerance towards parasites and to induce fibrosis in several examples of helminth infection [12]. However, opposite to the recognized role of Interleukin-10 [12], [13], little is known about TGF-*β* involvement in the pathophysiology of larval echinococcosis. Only preliminary studies are available in AE: Zhang et al. [14] showed that TGF-*β* was expressed in most lymphocytes of the periparasitic infiltrate in liver biopsies from AE patients. It was suggested that TGF-*β* may play a role in maintaining host tolerance against *E. multilocularis* growth by preventing T-cell cytotoxicity against the parasite [14]. In cystic echinococcosis (CE), immunostaining of TGF-*β* has also been shown at the periphery of hydatid cysts in the liver of patients [15]; and another study confirmed a progressive increase in the expression of mRNA of TGF-*β* in the liver of *E. granulosus*-infected BALB/c mice [16]. There is abundant evidence that TGF-*β*1, besides its role in immune tolerance, is an extremely potent inducer of the synthesis of procollagen and other extra-cellular matrix (ECM) components [17], [18], and has an essential role in the pathogenesis of liver fibrosis. The major signaling pathway for all TGF-*β* members is activated through ligand binding to a cell-surface receptor complex of type I and type II serine–threonine kinases receptors; and a group of intracellular signaling intermediates known as Smads is then phosphorylated. Phosphorylated Smads translocate to the nucleus where they function as transcription factors, initiating target gene transcription [19]. Smad4 is apparently common to all ligand-specific Smad pathways, and is a central mediator in TGF-*β* superfamily signaling [20]. Smad7, which is induced by TGF-*β* itself, forms part of an inhibitory feedback loop by binding to the intracellular domain of the activated TGF-*β* RI [21]–[24]. Because Smad7 is responsible for the fine-tuning of TGF-*β* signals [25], an aberrant expression of Smad7 might disrupt the balanced activity of TGF-*β* under physiological and pathophysiological conditions. However, although it may be crucial in the host-parasite interactions (Fig. 1), the relationship between the TGF-*β*/Smad pathway, and especially Smad7 expression, and clinical and/or pathological features of AE in experimental models as well as in humans has never been addressed.

The aims of this study were 1) to delineate the location of TGF-*β* and components of the TGF-*β* pathway in the periparasitic immune cells and in hepatocytes, close to and distant from the lesions in the liver; 2) to better understand the functioning of the TGF-*β*/Smad pathway, and its possible relationship with the development of liver fibrosis in the parasite’s hosts; 3) to further explore how TGF-*β* was secreted and regulated. For this purpose, and to get a comprehensive appraisal of TGF-*β* secretion and of its role in *E. multilocularis* infection, experimental AE in a mouse model of liver-targeted secondary AE [4] allowed us to study the time course of TGF-*β* expression as well as the dynamics of TGF-*β* signaling-related components, TGF-*β* RI, TGF-*β* RII, pSmad 2/3, Smad4 and Smad7, and to correlate them with the time course of the periparasitic infiltration by T-cell subpopulations, and to biochemical indicators of liver fibrosis, such as *α*-smooth muscle actin (*α*-SMA), and collagens I (COL I), and III (COL III). We also studied TGF-*β* and TGF-*β* signaling-related components in the liver of AE patients both at the protein and mRNA levels, in order to assess the situation at the late stage of infection in resistant hosts where immune tolerance and development of fibrosis are combined.

## Results

### Pathological Examination of the Livers Infected with *E. multilocularis*

In experimental mice, at the very early stage (2 and 8 days p.i.), in the surrounding of the metacestode injection site, lipid accumulation was observed in some hepatocytes (focal steatosis), and lymphocytes infiltrated the portal areas. No obvious change was found in the distant liver. From day 30 to day 90 after infection, at the periphery of the lesion, fibroblasts and inflammatory cells proliferated and an obvious increase of liver fibrosis was observed at the periphery of the lesion. There was no change in the areas distant from the lesion, except fibroblast and Kupffer cell proliferation, and an increased presence of lymphocytes in portal spaces. From day 180 to day 360, the typical granulomatous and fibrous periparasitic infiltrate of AE was fully established; in the liver, degenerating hepatocytes with atrophy and necrosis, as well as fibrous tissue development were observed in the areas immediately surrounding the granulomatous host response. Both fibroblasts and Kupffer cells proliferated in areas distant from the lesion. Mice in the control group at the same time-points showed normal hepatic histology (data not shown; available from reference [26]).

In AE patients, the liver lesions were similar to those observed in experimental mice at day 180 after infection, with the typical granulomatous and fibrous reaction surrounding parasite vesicles either active or degenerating. In the liver areas distant from the lesions, there was Kupffer cell proliferation, and lymphocytes infiltrated the portal areas. In the liver areas immediately surrounding the lesions, a few hepatocytes showed degeneration (data not shown).

### Expression of *α*-SMA, and Collagen I, III in the Livers Infected with *E. multilocularis*

**Figure 2:**
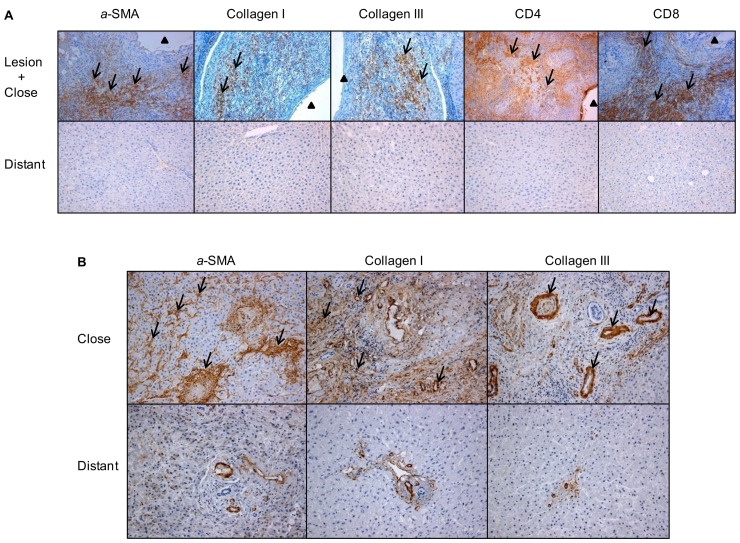
Immuno-histochemical expression of fibrosis markers in *E. multilocularis*-infected livers of experimental mice and AE patients, and of the periparasitic infiltration by CD4^+^ T and CD8^+^ T lymphocytes in the liver of experimental mice (arrow). A: In experimental mice. *α*-SMA: expression at day 8, in the cytoplasm of smooth muscle cells, hepatic stellate cells and myofibroblasts in the liver parenchyma; collagen I: expression at day 360, in the peri-parasitic granuloma as concentric bundles extending from the laminated layer of the parasitic vesicles to the border of the normal liver; collagen III: expression at day 360, in the peri-parasitic granuloma as concentric bundles extending from the laminated layer of the parasitic vesicles to the border of the normal liver, also present as dotted lines between the cells at the outer part of the granulomatous infiltrate and, occasionally, in the cytoplasm of round cells in the sinusoids of the surrounding liver; CD4^+^ T cells: expression at day 90, in the periparasitic infiltrate surrounding the metacestode; CD8^+^ T cells: expression at day 180, in the periparasitic infiltrate surrounding the metacestode. B: In AE patients. α-SMA: expressed in the extracellular matrix; collagen I expressed both in the extracellular matrix and hepatocytes; collagen III expressed in the extracellular matrix and hepatocytes. The arrowheads indicate the parasitic lesions in the liver of infected mice and human patients. Final magnification: 200×. ‘Lesion’: *E. multilocularis* metacestode and surrounding immune infiltrate; ‘Close’: liver parenchyma close to *E. multilocularis* lesion; ‘Distant’: liver parenchyma distant from *E. multilocularis* lesion.

**Figure 3:**
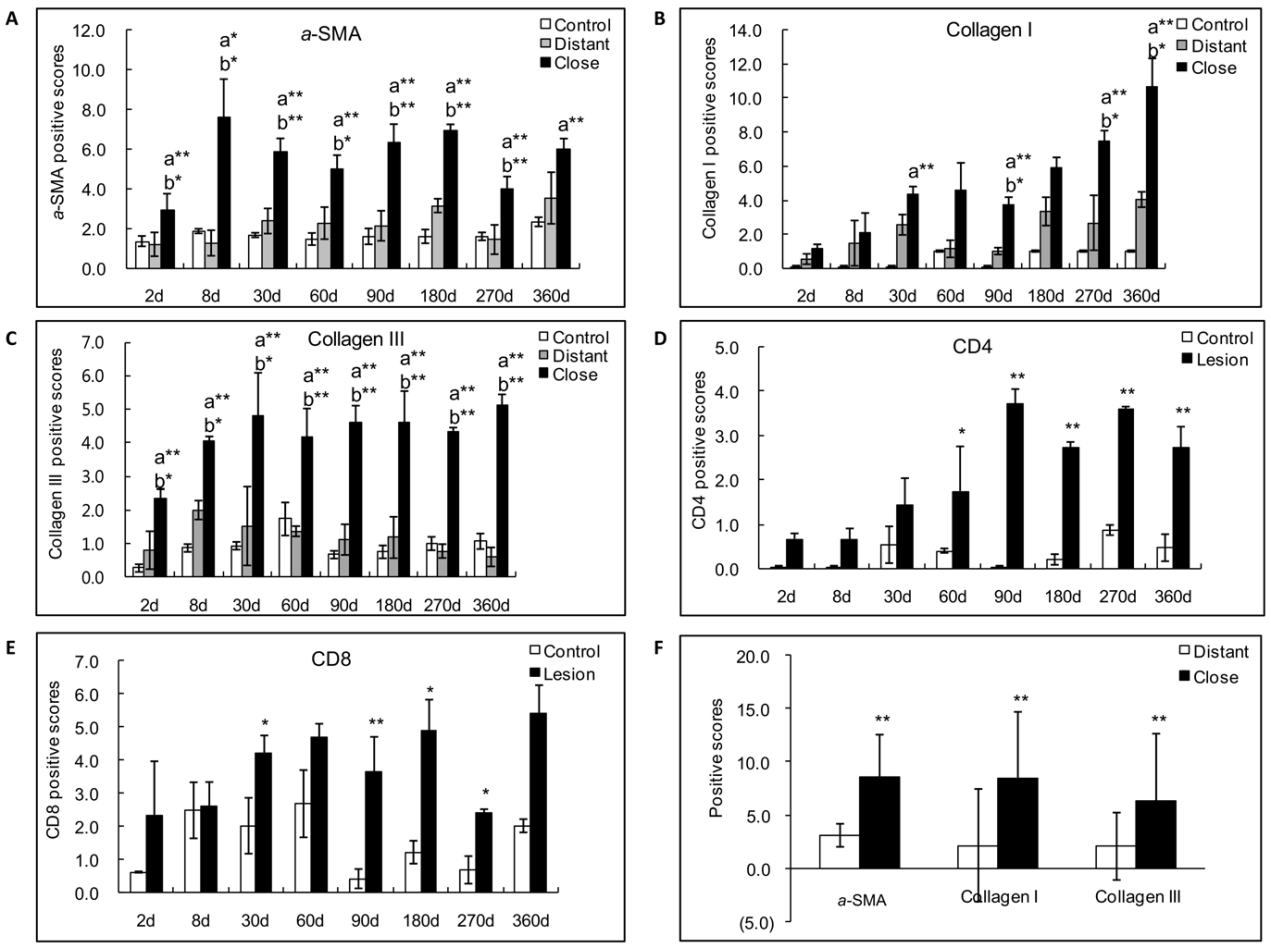
Semiquantitative expression of fibrosis markers in *E. multilocularis*-infected liver in experimental mice and in AE patients. Score for each marker expression was calculated from quantitative analysis of the histo-immunostaining using both staining intensity and the percentage of cells stained at a specific range of intensities (arrow) (see Materials and Methods section). A: Course of *α*-SMA expression in *E. multilocularis*-infected mice. B: Course of collagen I expression in *E. multilocularis*-infected mice; C: Course of collagen III expression in *E. multilocularis*-infected mice; D: Course of CD4^+^ T cell infiltration in *E. multilocularis*-infected mice; E: Course of CD8^+^ T cell infiltration in *E. multilocularis*-infected mice; F: Expression of fibrosis markers in the liver of AE patients. a: close versus control; b: close versus distant. *P<0.05; **P<0.01. ‘Control’, non-infected mice; ‘Lesion’: *E. multilocularis* metacestode and surrounding immune infiltrate; ‘Close’: liver parenchyma close to *E. multilocularis* lesion; ‘Distant’: liver parenchyma distant from *E. multilocularis* lesion.

In experimental mice, in the liver of control animals *α*-SMA expression was present in the cytoplasm of smooth muscle cells, i.e. restricted to the walls of most of the portal and central veins while there was nearly no staining in the liver parenchyma (Fig. 2). 180 days after *E. multilocularis* infection, *α*-SMA positive score was higher in infected than in control mice; distribution of *α*-SMA positive cells was diffuse in the liver parenchyma, suggesting a myofibroblastic differentiation of stellate cells in the liver (Fig. 3A).

In AE patients, in the liver areas close to lesions, there was a strong *α*-SMA immunostaining present in the ECM and *α*-SMA expression scores were significantly higher in the areas close to lesions compared to those distant from lesions (Fig. 2 and 3F).

In experimental mice, there was a marked difference between *E. multilocularis*-infected mice and control mice with regard to the nature and location of collagens in the liver. At all time-points, strong staining for Collagen I and III was present in the peri-parasitic granuloma as concentric bundles extending from the laminated layer of the parasitic vesicles to the border of the normal liver (Fig. 2). Collagen III was also present as dotted lines between the cells at the outer part of the granulomatous infiltrate and, occasionally, in the cytoplasm of round cells in the sinusoids of the surrounding liver (Fig. 2).

In AE patients, in the liver areas close to lesions, there was a strong Collagen I and III immunostaining in the ECM. Expression scores of Collagen I and Collagen III were significantly higher in the areas close to lesions compared to those distant from lesions (Fig. 3F).

### Infiltration by CD4^+^ and CD8^+^ T Cells in the Periparasitic Area in *E.multilocularis*-infected Mice

As the experimental model of AE allowed us to study the correlation, if any, between T lymphocyte infiltration in the liver and TGF-*β* expression over the time course of infection, CD4 and CD8 immunostaining was performed in the liver of mice; this was not performed in the liver of AE patients, since the time course could not be assessed. There was nearly no infiltration by CD4^+^ T cells nor by CD8^+^ T cells in the control groups whenever the time point after sham injection of saline in the liver (Fig. 2). In the periparasitic infiltrate surrounding the metacestode, in experimental infected mice, CD4^+^ T cells were present from day 60 to day 360. CD4 positive scores ranged from 0.6 to 3.7 and reached the peak point at day 90 (Fig. 3D). Infiltration by CD8^+^ T cells was expressed by scores which ranged from 2.3 to 5.4 and reached the peak point later than CD4^+^ T cells, at day 360 (Fig. 3E).

### Expression of TGF-*β*1 in the Livers Infected with *E. multilocularis*

#### Protein expression of TGF-β1

**Figure 4:**
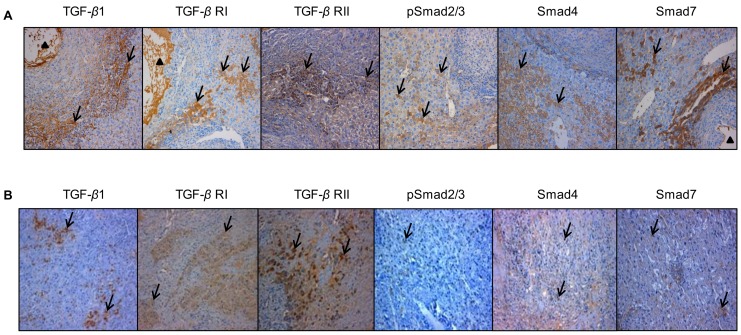
Immunohistochemical expression of the various components of the TGF-*β*/Smad pathway in the *E. multilocularis*-infected liver in experimental mice and in AE patients. A: In experimental mice. Expression of the various components of the TGF-*β*/Smad pathway at their peak of expression in the liver. TGF-*β*1: expression at day 90, in most of the immune cells in most of areas with inflammatory granulomas, in the cytoplasm of hepatocytes, endothelial cells of the hepatic sinusoids and fibroblasts; TGF-*β* RI and RII: expression at day 60, in the cytoplasm of lymphocytes and macrophages in the periparasitic infiltrate and in most of the hepatocytes, fibroblasts, and endothelial cells close to the periparasitic infiltrate; pSmad2/3: expression at day 30, in both the cytoplasm and nuclear of the hepatocytes; Smad4: expression at day 60, in both the cytoplasm and nuclear of the hepatocytes; Smad7: expression at day 90, in the cytoplasm of the hepatocytes. B: In AE patients. Specimen ‘Close’ was taken close to the parasitic lesions (0.5 cm from the macroscopic changes due to the metacestode/granuloma lesion), and Specimen ‘Distant’ was taken in the liver distant from the lesions (the non-diseased lobe of the liver whenever possible, or at least at 10 cm from the lesion). TGF-*β*1: expressed in most of the immune cells in most of areas with inflammatory granulomas, in the cytoplasm of hepatocytes, endothelial cells of the hepatic sinusoids and fibroblasts; TGF-*β* RI and RII: expressed in the cytoplasm of lymphocytes and macrophages in the periparasitic infiltrate and in most of the hepatocytes, fibroblasts, and endothelial cells close to the periparasitic infiltrate; pSmad2/3: expressed in both the cytoplasm and nuclear of the hepatocytes; Smad4: expressed in both the cytoplasm and nuclear of the hepatocytes; Smad7: expressed in the cytoplasm of the hepatocytes. The arrowheads indicate the parasitic lesions in the liver of infected mice and human patients. Final magnification: 200×.

**Figure 5:**
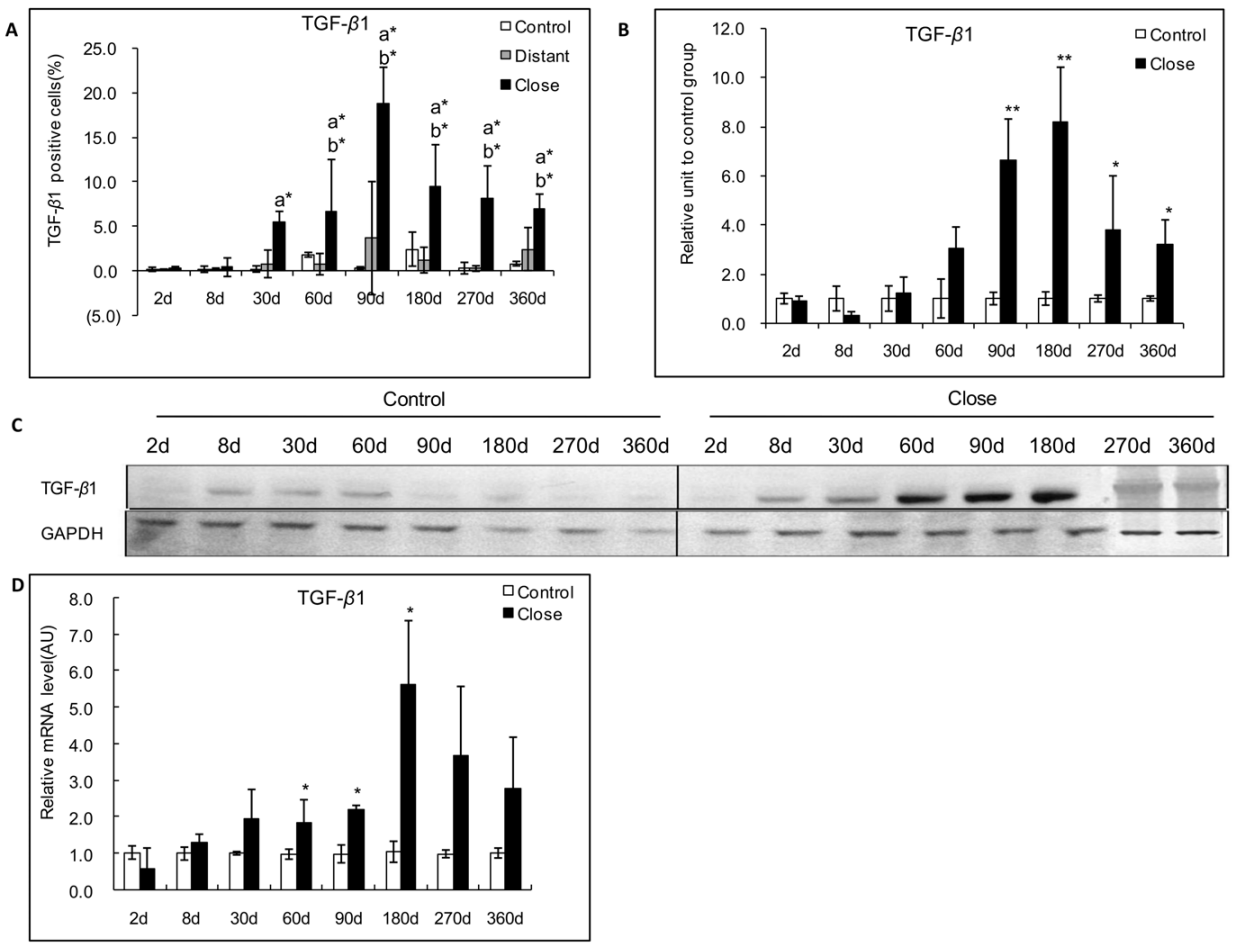
Course of TGF-*β*1 expression in the liver of experimental mice during *E. multilocularis* infection. A: Course of TGF-*β*1 expression observed by immune-staining in the liver from *E. multilocularis* infected mice, calculated as the percent of positive cells to the total number of counted cells (see Materials and Methods section). B: Relative amount of TGF-*β*1 calculated from semi-quantitative analysis of the Western Blot using densitometry. C: Representative example of the course of TGF-*β*1 protein measured by Western Blot. D: Course of TGF-*β*1 mRNA expression measured by real time RT-PCR. a: ‘close’ versus ‘control’; b: ‘close’ versus ‘distant’. *P<0.05; **P<0.01. ‘Control’, non-infected mice; ‘Lesion’: *E. multilocularis* metacestode and surrounding immune infiltrate; ‘Close’: liver parenchyma close to *E. multilocularis* lesion; ‘Distant’: liver parenchyma distant from *E. multilocularis* lesion. AU: arbitrary units; GAPDH: glyceraldehyde-3-phosphate dehydrogenase.

In experimental mice, a strong immunostaining for TGF-*β*1 was observed in the periparasitic infiltrate in most of areas with inflammatory granulomas from 30 days to 360 days p.i. In the liver area close to the parasitic lesions, a faint expression of TGF-*β*1 was observed in the endothelial cells at day 30; a marked expression was observed in endothelial cells of the hepatic sinusoids and in fibroblasts at day 60, as well as in endothelial cells of the hepatic sinusoids and in hepatocytes close to the parasitic lesions from 90 days to 360 days p.i. In the liver distant from the parasitic lesions, a faint staining for TGF-*β*1 was observed in the endothelial cells of hepatic sinusoids from 30 to 90 days p.i.; there was a moderate staining in endothelial cells of hepatic sinusoids from 180 to 360 days p.i., while a faint staining was observed in the hepatocytes from 90 and 360 days p.i. (Fig. 4 and 5A). An increased TGF-*β*1 expression measured using Western Blot in the liver of experimental infected mice was observed from day 2 (0.9-fold) to day 360 (3.2-fold); it peaked at day 180 (8.2-fold) after infection with *E. multilocularis,* then decreased to lower levels, albeit higher than in control mice until the end of follow-up; difference between experimental and control mice was significant at day 90, 180, 270 and 360 (*P*<0.05).

**Figure 6:**
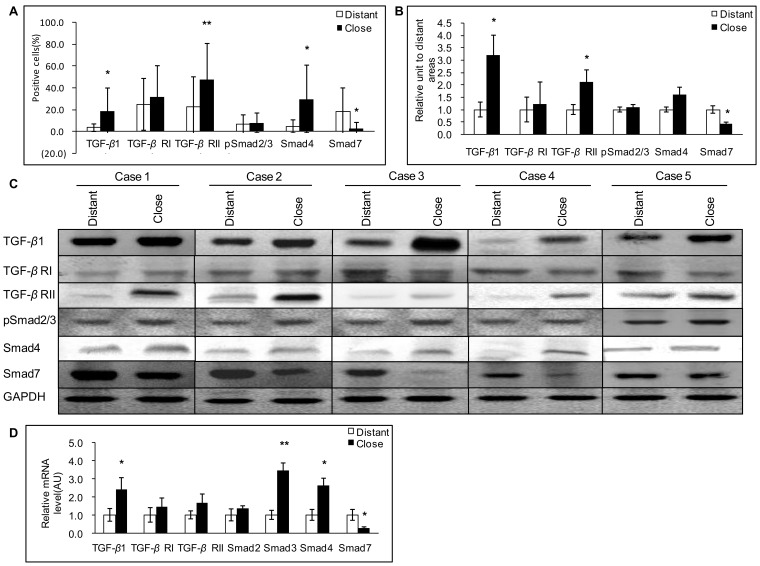
Expression of the various components of the TGF-*β*1/Smad pathway in the liver of AE patients. A: Expression of TGF-β1/Smads calculated as the percent of positive cells to the total number of counted cells after immunostaining (see Materials and Methods section). B: Relative amount of TGF-*β*1/Smads calculated from semi-quantitative analysis of the Western Blot using densitometry. C: Representative examples of Western Blot analyses performed on lysates from liver samples with antibodies that recognize TGF-*β*1, TGF-*β* RI, TGF-*β* RII, phosphorylated (p-) Smad2/3, Smad4 and Smad7. D: TGF-*β*1/Smads mRNA expression measured by real time RT-PCR. *P<0.05 versus control, **P<0.01 versus control. ‘Distant’: distant from lesion; ‘Close’: close to lesion; AU: arbitrary units; GAPH: glyceraldehyde-3-phosphate dehydrogenase.

In AE patients, as observed in the mouse model, a strong immunostaining for TGF-*β*1 was observed in most lymphocytes and macrophages in the periparasitic infiltrate, as well as in Kupffer cells, fibroblasts, and endothelial cells in hepatic sinusoids, especially around the granulomas, and in infiltrating immune cells of portal spaces (Fig. 4). In the non-infiltrated liver, faint staining with anti-TGF-*β*1 antibodies was observed in hepatocytes, even in those observed in areas distant from the parasitic lesions (Fig. 4). Percentage of TGF-*β*1 positive cells was higher in areas close to than distant from lesions (Fig. 6A), with an intensity gradient from the periparasitic areas to the distant liver, since TGF-*β*1 staining appeared stronger close to the granulomatous reaction (Fig. 4). Western Blot measurements of TGF-*β*1 also showed that protein levels of the cytokine were significantly higher in the liver tissue close to lesions than in that distant from lesions (Fig. 6B and C) (*P*<0.05).

#### Correlation with T cell subpopulations and fibrosis markers

TGF-*β*1 expression in the periparasitic infiltrate was highly positively correlated with CD4/CD8 ratio (r = 0.818) but not correlated with either CD4^+^ or CD8^+^ T cell scores, taken independently (Table 1).

**Table 1:** Results of the correlation analysis between TGF-*β*1 and CD4/CD8, CD4, CD8 positive cells in murine AE (from the histo-immunochemistry analysis).

		CD4/CD8	CD4	CD8
TGF-*β*1	Spearman’s rho	0.818*	0.639	−0.118
	Sig.	0.013	0.088	0.780
	N	8	8	8

Note: *P<0.05.

Spearman correlation coefficients indicated a positive correlation between TGF-*β*1 expression and *α*-SMA, Collagen I, and Collagen III expression scores (r = 0.628, *P* = 0.009; r = 0.836, *P*<0.001; r = 0.781, *P*<0.001 respectively) in the livers from day 90 to day 360 p.i. in experimental mice under study (Table 2). There was also a positive correlation between TGF-*β*1 expression and *α*-SMA, Collagen I, and Collagen III expression scores (r = 0.620, *P* = 0.001; r = 0.498, *P* = 0.013; r = 0.655, *P* = 0.001 respectively) in the livers from the 16 patients with AE under study (Table 3).

**Table 2:** Results of the correlation analysis between TGF-*β*1, Smad7 and liver fibrosis markers in murine AE (from the histo-immunochemistry analysis).

		α-SMA	Collagen I	Collagen III
TGF-β1	Spearman’s rho	0.628**	0.836**	0.781**
	Sig.	0.009	*P*< 0.001	*P*< 0.001
	N	8	8	8
Smad7	Spearman’s rho	−0.600	−0.853**	−0.316*
	Sig.	0.400	*P*< 0.001	0.684
	N	8	8	8

Note: *P<0.05,

** *P*<0.01.

**Table 3:** Results of the correlation analysis between TGF-*β*1, Smad7 and liver fibrosis markers in human AE (from the histo-immunochemistry analysis).

		α-SMA	Collagen I	Collagen III
TGF-β1	Spearman’s rho	0.620**	0.498**	0.655**
	Sig.	0.001	0.013	0.001
	N	16	16	16
Smad7	Spearman’s rho	−0.569**	−0.313	−0.463*
	Sig.	0.004	0.136	0.023
	N	16	16	16

Note: *P<0.05,

** *P*<0.01.

#### RNA expression of TGF-*β*1

In experimental mice, real-time RT-PCR showed an increase in TGF-*β*1 mRNA expression from day 8 to the end of follow-up, with a peak at day 180 after infection. TGF-*β*1 mRNA expression increased from 0.57-fold at day 2 to 5.37-fold at day 180 (Fig. 5D) compared to control mice. There was a significant difference between *E. multilocularis* infected and control group at the time points of 60-, 90- and 180-days p.i. (*P*<0.05).

In AE patients, real-time RT-PCR showed that TGF-*β*1 mRNA expression was significantly higher in the liver tissue close to lesions compared to that distant from lesions (Fig. 6D) (*P*<0.05).

### Expression of TGF-*β* RI and RII in the Livers Infected with *E. multilocularis*

#### Protein expression of TGF-*β* RI and RII

**Figure 7:**
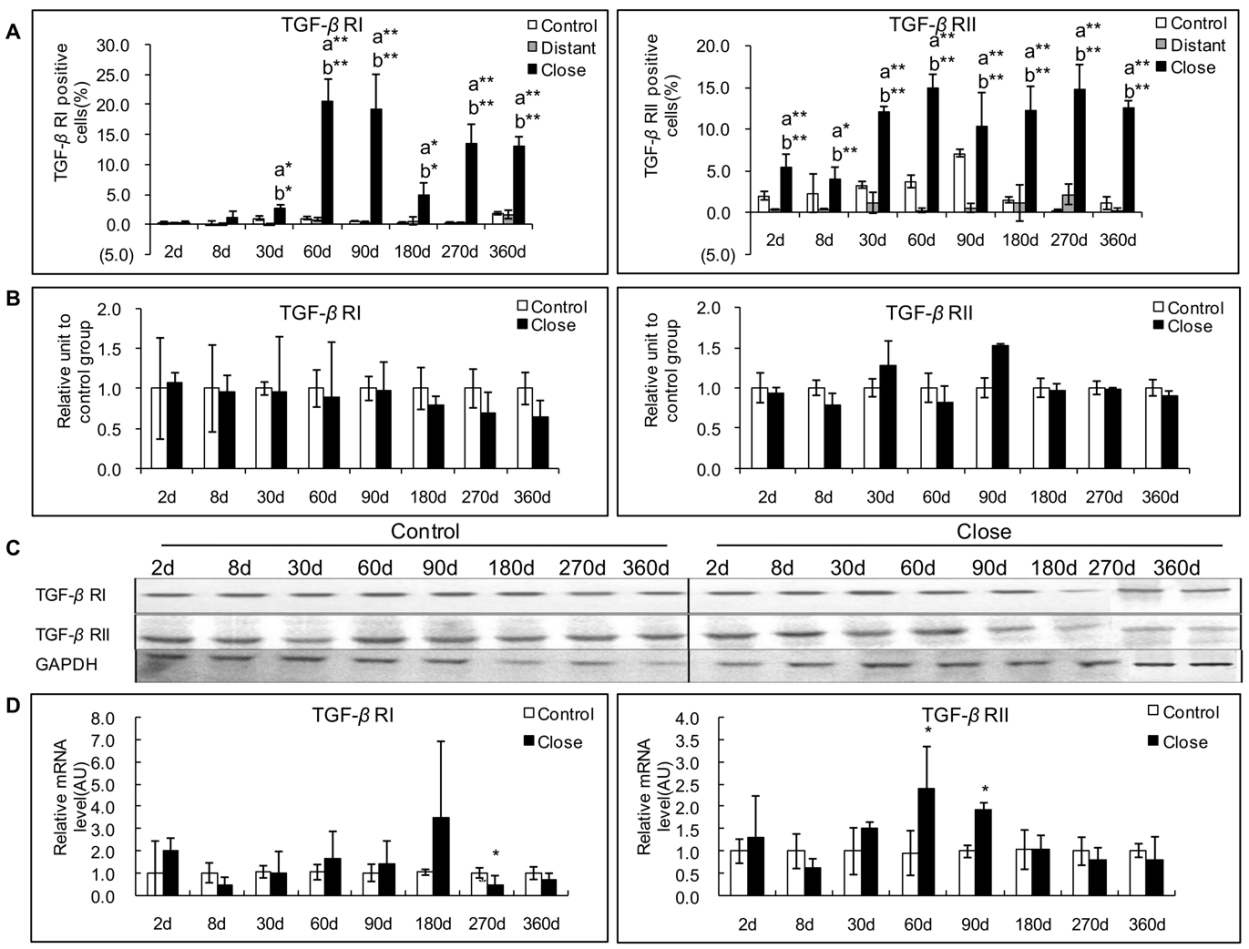
Course of TGF-*β*1 receptors (TGF-*β* RI and TGF-*β* RII) expression in the liver of mice during *E. multilocularis* infection in experimental mice. A: Course of TGF-*β* RI and RII expression observed by immune-staining in the liver from *E. multilocularis* infected mice compared to control mice, calculated as the percent of positive cells to the total number of counted cells (see Materials and Methods section). B: Relative amount of TGF-*β* RI and RII calculated from semi-quantitative analysis of the Western Blot using densitometry. C: Representative example of the course of TGF-*β* RI and RII protein measured by Western Blot in experimental mice. D: Course of TGF-*β* RI and RII mRNA expression measured by real time RT-PCR in experimental mice. a: ‘close’ versus ‘control’; b: ‘close’ versus ‘distant’. *P<0.05; **P<0.01. ‘Control’, non-infected mice; ‘Lesion’: *E. multilocularis* metacestode and surrounding immune infiltrate; ‘Close’: liver parenchyma close to *E. multilocularis* lesion; ‘Distant’: liver parenchyma distant from *E. multilocularis* lesion. AU: arbitrary units; GAPDH: glyceraldehyde-3-phosphate dehydrogenase.

In experimental mice, TGF-*β* RI immunostaining was observed in the cytoplasm of lymphocytes and macrophages in the periparasitic infiltrate, and in most of the hepatocytes, fibroblasts, and endothelial cells in the liver close to the periparasitic infiltrate; no positive staining was observed in the control liver sections (Fig. 4). Positive cells ranged from 0.25% to 20.5% and reached a peak at day 60 p.i. In the liver distant from the parasitic lesions, a very faint staining for TGF-*β* RI was observed in the endothelial cells of hepatic sinusoids from 30 to 90 days p.i., a faint staining was observed in the hepatocytes from 60 to 360 days p.i. and in endothelial cells of hepatic sinusoids from 180 to 360 days p.i. (Fig. 7A). There was a significant difference between *E. multilocularis*-infected and control groups, close to lesion and distant from lesion at all time-points since day 30 (*P*<0.05, Fig. 7A). However, Western Blot results could not show a significant difference in the protein levels of TGF-*β* RI and TGF-*β* RII in infected versus control mice during the whole time course of *E. multilocularis* infection. TGF-*β* RII immunostaining was observed in the same cells as TGF-*β* RI in infected mice (Fig. 4). Positive cells ranged from 4.0% to 15.0% and reached a peak at day 60. There was a significant difference between *E. multilocularis* infected and control groups, close to lesion and distant from lesion at all time-points (*P*<0.05, Fig. 7A).

In AE patients, expression of TGF-*β* RI differed markedly between patients and taking all 16 patients into account, there was no significant difference between the positive cells for TGF-*β* RI in areas close to and distant from lesions (Fig. 6A). Similarly, Western Blot results showed that TGF-*β* RI protein levels were not different in the liver tissue close to lesions compared to that distant from lesions (Fig. 6B and C). There was no significant difference either between the expression of TGF-*β* RII in areas close to and distant from lesions (Fig. 6B and C). However, compared with the areas distant from the parasitic lesions, the areas close to lesions displayed a stronger staining for TGF-*β* RII protein both at the cell membrane and in the cytoplasm (Fig. 4). Western Blot results showed that TGF-*β* RII protein levels were significantly elevated in the liver tissue close to lesions compared to that distant from lesions (Fig. 6B and C).

#### mRNA expression of TGF-β RI and RII

In experimental mice, an increased TGF-*β* RI mRNA expression was observed in infected mice at day 60 to day 180, which peaked at day180. *E.multilocularis* infection increased TGF-*β* RI mRNA expression from 0.43-fold at day 8 to 3.48-fold at day 180 (Fig. 7D). There was a significant difference between *E. multilocularis* infected and control groups at day 270 p.i. (*P*<0.05); however at that time, mRNA expression was lower in infected mice, despite a marked increase in the expression of the receptor as measured by immunostaining. Increased TGF-*β* RII mRNA expression was observed from day 30 to 90 and peaked at day 60 after infection. *E.multilocularis* infection increased TGF-*β* RII mRNA expression from 0.70-fold at 8 days to 2.52-fold at 60 days (Fig. 7D). The difference between *E. multilocularis*-infected and control mice was significant at day 60 and 90 (*P*<0.05).

In AE patients, there were no significant differences in TGF-*β* RI as well as TGF-*β* RII mRNA levels measured by real-time RT-PCR in the liver close to and distant from the lesions (Fig. 6D).

### Phosphorylation of Smad2/3 and Expression of Smad4 in the Livers Infection with *E. multilocularis*

#### Protein expression of phosphorylated Smad 2/3

**Figure 8:**
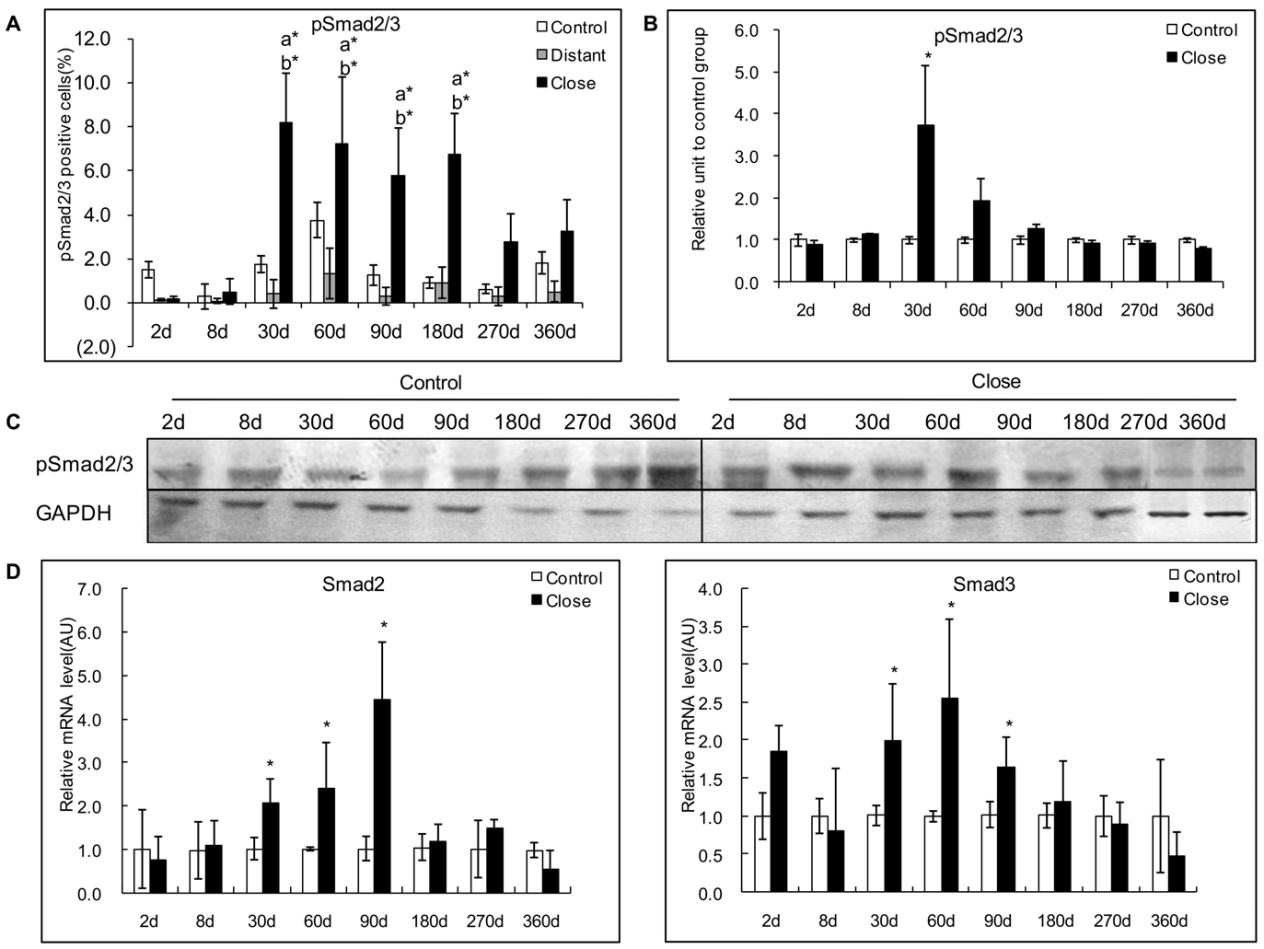
Course of pSmad2/3 expression in the liver of mice during *E. multilocularis* infection in experimental mice. A: Course of pSmad2/3 expression observed by immune-staining in the liver from *E. multilocularis* infected mice compared to control mice, calculated as the percent of positive cells to the total number of counted cells (see Materials and Methods section). B: Relative amount of pSmad2/3 calculated from semi-quantitative analysis of the Western blot using densitometry. C: Representative example of the course of pSmad2/3 protein measured by Western Blot in experimental mice. D: Course of Smad2 and Smad3 mRNA expression measured by real time RT-PCR in experimental mice. a: ‘close’ versus ‘control’; b: ‘close’ versus ‘distant’. *P<0.05; **P<0.01. ‘Control’, non-infected mice; ‘Lesion’: *E. multilocularis* metacestode and surrounding immune infiltrate; ‘Close’: liver parenchyma close to *E. multilocularis* lesion; ‘Distant’: liver parenchyma distant from *E. multilocularis* lesion. AU: arbitrary units; GAPDH: glyceraldehyde-3-phosphate dehydrogenase.

In experimental mice, pSmad2/3 was most usually expressed in the cytoplasm of the hepatocytes; very little nuclear expression was observed. In infected mice, immunostaining displayed a patchy distribution, and strong staining was observed in those hepatocytes which were close to the periparasitic infiltrate (Fig. 4). Conversely, no or a faint staining was observed in the liver distant from the parasitic lesions and in the liver from control mice. The positive cells of pSmad 2/3 ranged from 0.15% to 8.2% in the liver close to lesion, and reached a peak at day 30 and day 60 after infection. There was a significant difference between *E. multilocularis* infected and control groups, close to lesion and distant from lesion at day 30, 60, 90 and 180 (*P*<0.05, Fig. 8A). Western Blot measurements showed that there was no difference either in the phosphorylation of Smad2/3 protein in the areas close to lesions compared to those distant from lesions (Fig. 8B and C).

In AE patients, immunostaining of pSmad2/3 displayed a patchy distribution (Fig. 4), however non-related to the lobular structure of the liver and/or to the distance to the parasitic lesions. There was no significant difference between the positive cells of pSmad2/3 in areas close to and distant from lesions (Fig. 6A). Western Blot measurements showed that there was no difference either in the phosphorylation of Smad2/3 protein in the areas close to lesions compared to those distant from lesions (Fig. 6B and C).

#### mRNA expression of Smad2 and 3

In experimental mice, increased Smad2 and Smad3 mRNA expression was observed from day 30 to day 90. Smad2 mRNA expression peaked at day 90 and ranged from 0.8-fold at day 2 to 4.4-fold at day 90 (Fig. 8D). There was a significant difference between *E. multilocularis* infected and control groups at day 30 (*P*<0.05). Smad3 mRNA expression peaked at day 60 and ranged from 2.6-fold at day 60 days to 0.5-fold at day 360 (Fig. 8D). There was a significant difference of between *E. multilocularis* infected and control groups at day 60 (*P*<0.05).

In AE patients, there were no significant differences in Smad2 mRNA levels measured by real-time RT-PCR (Fig. 6D). However, mRNA levels of Smad3 were significantly higher in tissue samples close to lesions compared to those distant from lesions (Fig. 6D).

#### Protein expression of Smad4

**Figure 9:**
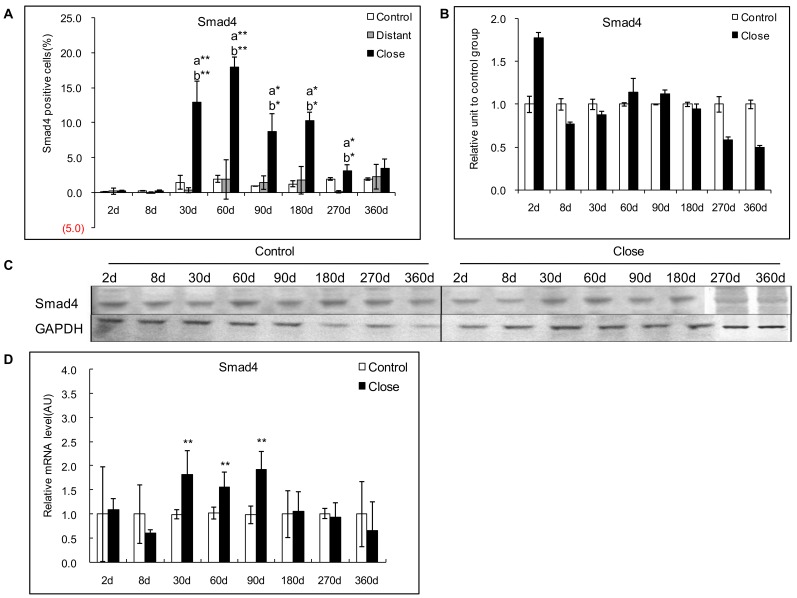
Course of Smad4 expression in the liver of mice during *E. multilocularis* infection in experimental mice. A: Course of Smad4 expression observed by immune-staining in the liver from *E. multilocularis* infected mice compared to control mice, calculated as the percent of positive cells to the total number of counted cells (see Materials and Methods section). B: Relative amount of Smad4 calculated from semi-quantitative analysis of the Western blot using densitometry. C: Representative example of the course of Smad4 protein measured by Western Blot in experimental mice. D: Course of Smad4 mRNA expression measured by real time RT-PCR in experimental mice. a: ‘close’ versus ‘control’; b: ‘close’ versus ‘distant’. *P<0.05; **P<0.01. ‘Control’, non-infected mice; ‘Lesion’: *E. multilocularis* metacestode and surrounding immune infiltrate; ‘Close’: liver parenchyma close to *E. multilocularis* lesion; ‘Distant’: liver parenchyma distant from *E. multilocularis* lesion.AU: arbitrary units; GAPDH: glyceraldehyde-3-phosphate dehydrogenase.

In experimental mice, immunohistochemical study of Smad4 protein revealed higher cytoplasmic and nuclear staining of hepatocytes in areas close to lesions compared with areas distant from lesions. Distribution of Smad4 expression was similar to that of pSmad2/3 in its location, but more diffuse (Fig. 4). Positive cells for Smad4 ranged from 0.2% to 18.0% in the liver close to lesion, and reached a peak at day 60 p.i.There was a significant difference between *E. multilocularis*-infected and control groups, close to lesion and distant from lesion at day 30, 60, 90, 180, and 270 (*P*<0.05, Fig.9A). However, Western Blot analysis of Smad4 expression did not show any difference between infected and control mice, as well as between the liver close to and distant from the lesions, all over the time course (Fig. 9B and C).

In AE patients, immunohistochemical staining for Smad4 protein in the liver revealed cytoplasmic and nuclear staining of hepatocytes, with a homogenous distribution among cells; positive cells were higher in the areas close to lesions compared with those distant to lesions. Intensity of nuclear staining of Smad4 protein was noticeably higher in the areas close to the lesions (Fig. 4). Western Blot results also confirmed that Smad4 protein levels were significantly higher in the liver parenchyma close to lesions compared to that distant from lesions (Fig. 6B and C).

#### mRNA expression of Smad4

In experimental mice, increased Smad4 mRNA expression was observed from day 30 to day 90. Smad4 mRNA expression peaked at day 90 and ranged from 0.6-fold at day 8 to 1.9-fold at day 90 (Fig. 9D). There was a significant difference between *E. multilocularis* infected and control mice at day 90 (*P*<0.05).

In AE patients, the “lesions to periphery gradient” observed for the protein expression was also demonstrated at the mRNA level, as measured by real-time RT-PCR, with higher expression close to the lesions (Fig. 6D).

### Expression of Smad 7 in the Livers Infected with *E. multilocularis*

#### Protein expression of Smad7

**Figure 10:**
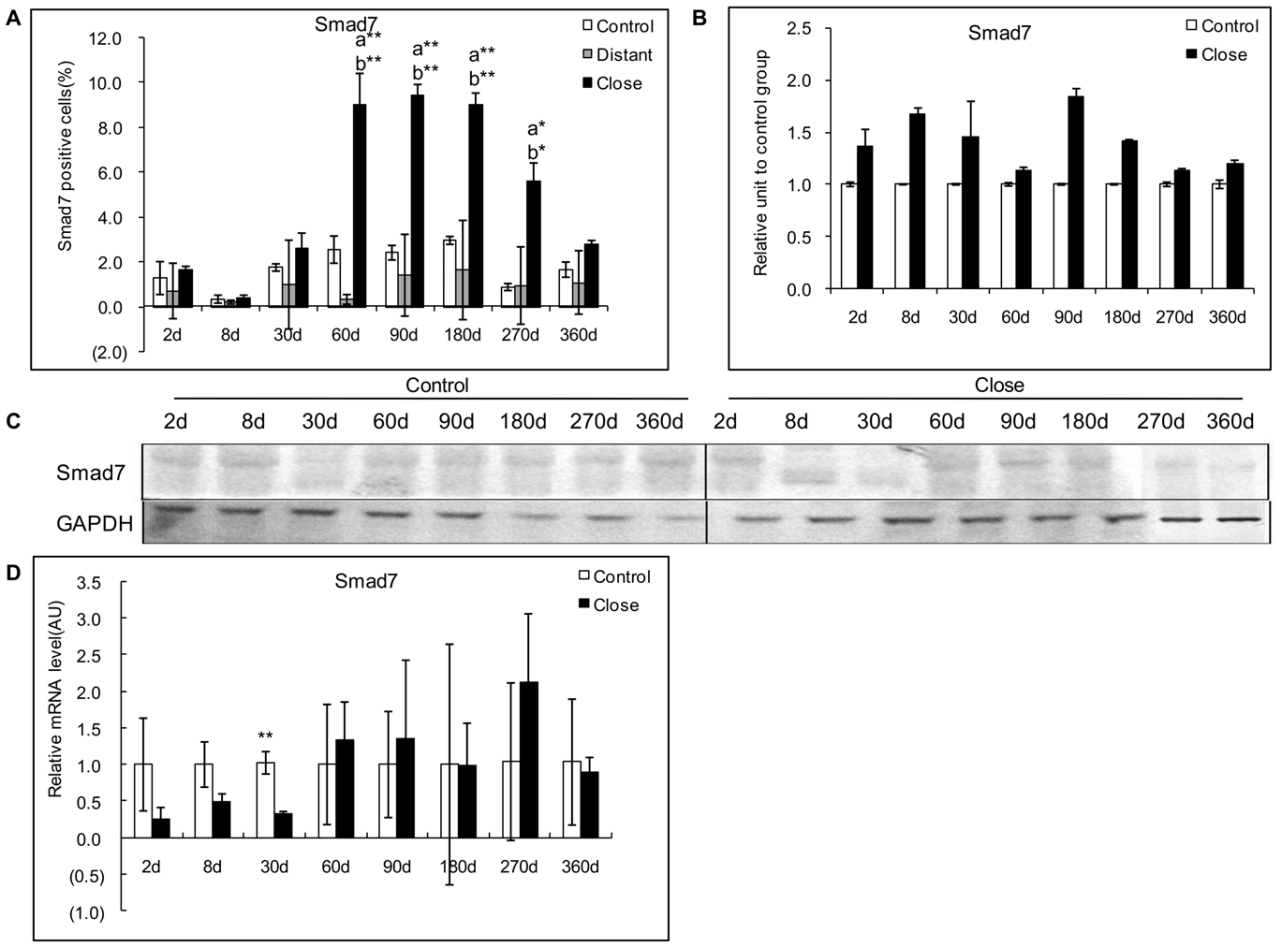
Course of Smad7 expression in the liver of mice during *E. multilocularis* infection in experimental mice. A: Course of Smad7 expression observed by immune-staining in the liver from *E. multilocularis* infected mice compared to control mice, calculated as the percent of positive cells to the total number of counted cells (see Materials and Methods section). B: Relative amount of Smad7 calculated from semi-quantitative analysis of the Western blot using densitometry. C: Representative example of the course of Smad7 protein measured by Western Blot in experimental mice. D: Course of Smad7 mRNA expression measured by real time RT-PCR in experimental mice. a: ‘close’ versus ‘control’; b: ‘close’ versus ‘distant’. *P<0.05; **P<0.01. ‘Control’, non-infected mice; ‘Lesion’: *E. multilocularis* metacestode and surrounding immune infiltrate; ‘Close’: liver parenchyma close to *E. multilocularis* lesion; ‘Distant’: liver parenchyma distant from *E. multilocularis* lesion. AU: arbitrary units; GAPDH: glyceraldehyde-3-phosphate dehydrogenase.

In experimental mice, Smad7 immunostaining was mostly present in the cytoplasm of the hepatocytes, with a varying intensity throughout the liver, higher in the areas close to the lesion than in those distant from the lesion (Fig. 4). A faint staining was observed in the hepatocytes from 30 to 360 days in the areas distant from the lesion and in the control group (Fig. 10A). Smad7 positive cells in the hepatic cells ranged from 0.40% to 9.45% and reached a peak at 90 days. There was a significant difference between *E. multilocularis* infected and control groups, close to lesion and distant from lesion at 60 days, 90 days, 180 days and 270 days p.i. (*P*<0.05, Fig. 10A). Western Blot results showed that there was no change in Smad7 expression between *E. multilocularis* infected and control mice during the whole time course of *E. multilocularis* infection (Fig. 10B and C).

In AE patients, Smad7 immunostaining was mostly present in the cytoplasm of the hepatocytes, with a varying intensity throughout the liver. Opposite to the decreasing gradient from the lesions to the distant parenchyma observed with most of the other components, the expression scores of Smad7 expression were lower in the areas close to lesions than in those distant from the lesions (Fig. 4B). Western Blot results also showed that Smad7 protein levels were significantly lower in the liver parenchyma close to lesions compared to that distant from lesions (Fig. 6B and C).

#### Correlation with T cell subpopulations and fibrosis markers

Spearman correlation coefficients indicated a negative correlation between Smad7 expression and α-SMA, Collagen I, and Collagen III expression scores which was significant for Collagen I and Collagen III (r = −0.853, *P*<0.01; and r = −0.316, *P*<0.05 respectively) in the livers from day 90 to day 360 p.i. in experimental mice under study (Table 2). There was also a negative correlation between Smad7 expression and α-SMA, Collagen I, and Collagen III expression scores, which was significant for α-SMA and Collagen III (r = −0.569, *P* = 0.01; and r = −0.463, *P* = 0.05, respectively) in the livers from the 16 patients with AE under study (Table 3).

#### mRNA expression of Smad7

In experimental mice, Smad7 mRNA expression was significantly higher in *E. multilocularis*-infected than in control mice at day 30 (*P*<0.01) (Fig. 10D).

In AE patients, lower Smad7 mRNA levels close to the lesions, as measured by real-time RT-PCR, further confirmed the reverse gradient from the lesions to the periphery observed at the protein level (Fig. 6D).

#### Correlation with fibrosis markers

There was a significant negative correlation between Smad7 and Collagen I expression scores in experimental mice under study (r = −0.853, *P*<0.001) (Table 2). There was also a significant negative correlation between Smad7 and α-SMA and Collagen III expression scores (r = −0.569, *P* = 0.004; r = −0.463, *P* = 0.023 respectively) (Table 3) in the liver of the 16 AE patients under study.

## Discussion

**Figure 11:**
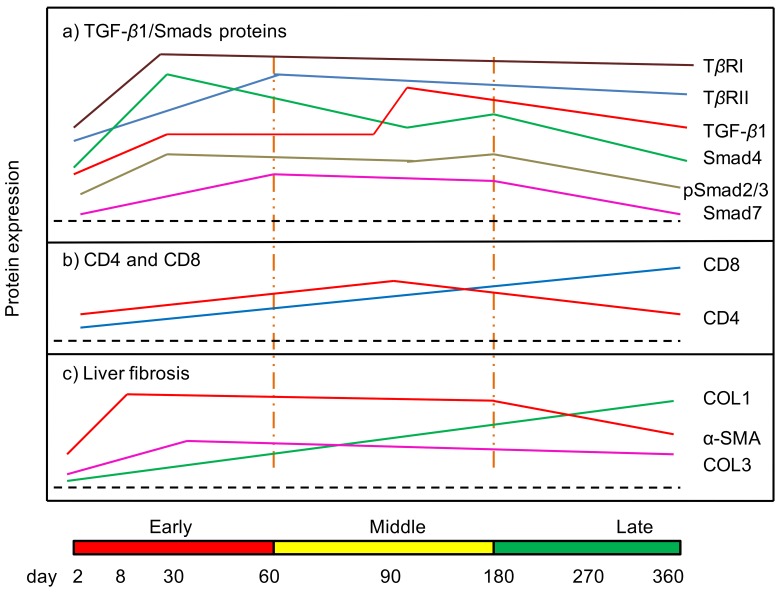
Course of the changes in the protein expression of TGF-β1/Smads (a), T cell subpopulation CDs (b) and liver fibrosis markers (c) during the process of *E. multilocularis*-induced liver injury in mice.

Despite the major potential role attributed to TGF-*β* in the tolerance and the fibrosis processes in AE, only one study until now reported that TGF-*β* was expressed in the periparasitic infiltrate in liver biopsies from a patient with AE [14]; however, quantified expression of TGF-*β* protein and mRNA was never studied, and neither the presence of TGF-*β* receptors nor that of components of the TGF-*β* metabolic pathway were ever looked for in *E. multilocularis*-infected livers. In the present study, both in humans and in the longitudinal study of experimental *E. multilocularis* infection model, we confirmed that TGF-*β* and members of its pathway were actually present in *E. multilocularis*-infected livers (Fig. 11). We could show the expression of TGF-*β* in most lymphocytes and macrophages of the periparasitic infiltrate as well as in the liver parenchyma, even distant from the parasitic lesion. Phenotypic study of cells within the periparasitic granuloma also confirmed that CD4^+^ T cells represented the major population of T cells at the beginning of the infection and that this sub-population was progressively replaced by CD8^+^ T cells [9], and this change of CD4/CD8 ratios could contribute to maintain TGF-*β*1 secretion. TGF-*β* receptors were also expressed at the membrane of most cells in the periparasitic infiltrate and in the liver parenchyma from early to late stage post *E. multilocularis* infection. Expression of the receptors suggested that the markedly elevated levels of TGF-*β*1 present in *E. multilocularis*-infected liver, were functional to regulate the activities of immune cells as well as hepatocytes and cells involved in fibrosis. This was confirmed by the changes also observed in various Smad components of the TGF-*β* pathway, with usually a marked increase since the middle stage of the chronic phase of the disease in *E. multilocularis* infected mice, which suggested an activation of the Smad cascade and thus an activation of the signal transduction of TGF-*β*1. Expression of the receptors and of Smads and phosphorylation of Smad 2/3 in the liver of human patients with hepatic AE, with various types of gradient in the liver depending on the cascade component, confirmed the significant activation of the system at the middle/late stage of *E. multilocularis* infection.

Fibrosis is a hallmark of AE, leading to a complete disappearance of the liver parenchyma in the periparasitic area, and to fibrosis in portal spaces. Fibrosis protects the host against the parasitic growth, but at the same time it distorts the liver parenchyma, contributes to bile duct and vessel obstruction and can lead to secondary biliary cirrhosis [5], [7]. The irreversible acellular keloid scar-like fibrosis observed in AE is the ultimate result of cytotoxic and fibrogenetic events related to the immune response of the host which are taking place initially in the granulomatous area surrounding the young parasite larvae [13]. Previous observations in experimental models of AE have suggested that progression of fibrosis in AE involves an early deposition of type III collagen pro-peptide and type III collagen at the periphery of the granulomas, and a subsequent remodeling of fibrosis with bundles of type I collagen in the periparasitic central area [4]. Stellate cell–derived myofibroblasts have been observed in AE liver, both in humans [7] and in the experimental mouse model [4]. It was noted that in some regions of the liver where the parenchyma was totally replaced with dead parasitic lesions and fibrosis, HSC were the only cellular remnants present [7]. We confirmed that *α*-SMA, a specific cell marker for MFB, as well as type I and III collagens, were highly expressed in tissues surrounding AE lesions; the expression of collagen I increased steadily through the course of the infection, whereas collagen III rapidly reached its maximum level of expression at day 8; this sequence of events, which is usual in fibrotic processes (collagen III being produced quickly by fibroblasts before collagen I is synthesized) was already noticed in the first studies on AE fibrosis in the experimental model; in humans, as well as in mice at later stages, location of collagen III in areas of recent larval development supported this sequence [4], [7].

The positive correlation we found between their expression and expression of TGF-*β*1, both in the experimental model and in human livers, is an indirect argument for a significant role of this cytokine in AE fibrosis. The major peak of TGF-*β*1 at the middle stage of infection in experimental animals, and its expression in AE patients who are diagnosed at a similar stage, suggest that although lower levels may initiate immune tolerance as early as the early stage, the cytokine becomes prominent later, when both maintenance of the tolerance state and development of fibrosis are at stake (Fig. 11). Several cytokines are involved in fibrosis development [27], [28]. The role of pro-inflammatory cytokines, and especially tumor necrosis factor (TNF-*α*), in the protection of the host against *E. multilocularis* has been demonstrated, and it is likely that they act at least in part through the development of fibrosis [29]. In human livers with hepatic AE, the mRNAs of pro-inflammatory cytokines, interleukin (IL)-1*β*, IL-6, and TNF-*α* have been found in macrophages located at the periphery of granulomas, in those areas which were shown to be at the initiation of fibrogenesis [30]. IL-12, which inhibits the development of the parasitic vesicles after *E. multilocularis* infection, was also shown to induce a fast development of peri-vesicle fibrosis [31]. However, TGF-*β* is probably the most decisive cytokine and HSCs the most significant cells involved in liver fibrosis [32]; and the involvement of TGF-*β* and HSCs in the development of the fibrosis in other liver parasitic diseases, such as schistosomiasis, has been well documented [33], [34]. During the development of chronic liver injury, including inflammation, fibrosis and regeneration, TGF-*β*1 plays a prominent role in stimulating liver fibrogenesis by MFBs derived from HSCs. TGF-*β*1 can be secreted by Kupffer cells, biliary cells, infiltrated inflammatory cells, and the HSC themselves; it inhibits hepatocyte proliferation, induces hepatocyte apoptosis, and activates HSC to differentiate into MFBs and secrete ECM components, including collagens, acting via both paracrine and autocrine pathways; TGF-*β*1 also inhibits ECM degradation and enhances accumulation of ECM in the liver [35]. Our results highly suggest that TGF-*β* and its signaling pathway are in the position to play this major role regarding fibrosis in AE.

The Smad family of proteins mediates signaling from the TGF-*β* R to the nucleus. In the current study, there was an increased expression of TGF-*β* R, Smad3 mRNA, and especially of Smad4 which is a central mediator in TGF-*β* superfamily signaling [20]. A few discrepancies between RNA expression and the amount of protein regarding TGF-*β* R and Smads may be explain by post-transcriptional events, and deserve further studies, since such events could be caused by parasite components. On the other hand, information given by immunostaining and Western Blot analysis is different, albeit complementary, since the type and microenvironment of the producing cells may compensate otherwise lower amounts of the protein. Our study showed that expression of Smad4 was higher in areas surrounding lesions than in distant liver in the patients with AE. Smad7, which is induced by TGF-*β* itself, is responsible for the fine-tuning of TGF-*β* signals [36]. It prevents the phosphorylation of Smad proteins, associates with ubiquitin ligases involved in TGF-*β* R-degradation, and acts as a transcriptional repressor inhibiting Smad-dependent promoter activation [37]. In physiological situations, its increase decreases the phosphorylation of Smad2/3, and thus decreases TGF-*β* functions. In chronic hepatic injury, the expression of Smad7 is paradoxically decreased [38]; as a result, TGF-*β* signal transduction cannot be effectively inhibited, and TGF-*β* functions are enhanced. An aberrant expression of Smad7 may thus disrupt the balanced activity of TGF-*β* under pathophysiological conditions. The low expression of Smad7 in the areas surrounding the lesions and its negative correlation with *α*-SMA and Collagen III highly suggest that in AE too the normal feed-back loop might not work properly, and that fibrosis might be permanently activated through that mechanism. As TGF-*β* is likely to be crucial to maintain the immune tolerance state and T-reg generation/function essential to the parasite, *E. multilocularis* could be responsible for the paradoxical decrease of Smad7 in the periparasitic granuloma and nearby liver; this might be one of the mechanisms for the early induction of immune tolerance and for the progression from chronic hepatic injury to hepatic fibrosis during *E. multilocularis* infection. All results obtained in the mouse model however do not fully support an essential role for the inhibitory Smad7 feed-back loop: Smad7 was indeed high in the middle stage of *E. multilocularis* infection, and Smad7 expression negatively correlated globally with expression of collagen I and III in infected mice, but this elevation did not seem to markedly decrease pSmad2/3, and Smad4 expression; the light decrease of these components at day 90, could be an indication of its partial intervention; other mechanisms of regulation of the TGF-*β* pathway at that crucial stage of the disease, to maintain a high level of activity of the pathway are thus likely. In fact, TGF-*β* also induces other non-SMAD signaling pathways, which include activation of several MKKs (MAP kinase Kinase) and MEKs (MAPK/ERK Kinase) pathways (including JNK/SPAK, p38, and ERK1/2) through upstream mediators RhoA, Ras, TAK1 (TGF-βActivated Kinase), TAB1 (TAK1 Binding Protein); and the proteins XIAP (Xenopus Inhibitor of Apoptosis), HPK1 (Haematopoietic Progenitor Kinase-1) are also involved in this link [39]. Thus, TGF-*β* itself or its receptors, more than down-stream Smads, represent an attractive target for the development of therapeutics that simultaneously attack the pathogen and its micro-environment, the pleiotropic nature of TGF-*β* signaling, its role in tissue homeostasis and its dual role in pathogenesis present unique challenges that must be considered in pre-clinical and clinical drug development programs.

In preliminary *in vitro* studies (data not shown) we observed a secretion of TGF- *β*1 and an activation of the TGF-*β* pathway in rat hepatocyte cultures incubated with vesicle fluid of parasitic origin, in the absence of inflammatory cells, thus of immune cell-related cytokines. This is an intriguing finding which reinforces the hypothesis of a “cross-talk” between the parasitic larva and its host, already provided by a number of observations which suggested that the larval development of *E. multilocularis* is triggered by cell signaling originating from the intermediate host [40], [41] and that *E. multilocularis* metacestode was thus able to “sense” host factors, which may result in an activation of the parasite metabolic pathway cascades [42]. Conversely, the parasite might also influence signaling mechanisms of host cells through the secretion of various molecules which might bind to host cell surface receptors or to the temporary storage of host-derived molecules in the vesicle fluid. Such interactions could contribute to immunomodulatory activities of *E. multilocularis,* to pathological consequences on the host’s tissues, and/or be involved in mechanisms of organotropism [14]. In our previous study, a significant influence of *E. multilocularis* metacestode on the activation of MAPKs signalling pathways was found in the liver cells both in vivo in infected patients and in vitro in cultured rat hepatocytes [43]. A recent study has also provided evidence for the induction of apoptosis in host DC through *Echinococcus* E/S-products of early infectious stages of *E. multilocularis* [44]. These observations suggest that parasitic components, and not only factors from host origin, are actually acting on the host [44]. Further studies are, however, necessary to determine the parasite and/or host components actually involved in the activation of the TGF-*β*/Smad pathway.

## Materials and Methods

### Ethics Statement

The clinical investigation has been conducted according to the principles expressed in the Declaration of Helsinki. For research involving human participants, informed written consent has been obtained from the patients, as part of a research project approved by the Ethical Committee of First Affiliated Hospital of Xinjiang Medical University (20080812-5). The animal study was performed in strict accordance with the recommendations in the Guide for the Care and Use of Laboratory Animals. The protocol was approved by the Animal Care and Use Committee and the Ethical Committee of First Affiliated Hospital of Xinjiang Medical University (20081205-2). All surgery was performed under sodium pentobarbital anesthesia, and every effort was made to minimize suffering.

### Experimental Design, Tissue Sampling and Histological Examination

#### Experimental animals

One hundred and twenty pathogen-free female BALB/c mice (8–10-week old) were housed in cages with a 12-h light/dark cycle and provided with rodent chow and water. BALB/c mice were infected by *E. multilocularis* and tissue samples were collected and detected as previously described [26], [45]. For each autopsy time-point, ten experimentally infected mice were used in *E*. *multilocularis* group (n = 10) and compared with five control mice (n = 5), which received an intra-hepatic injection of 0.1 mL of saline in the anterior liver lobe using the same surgical procedure. Mice were killed at 2, 8, 30, 60, 90, 180, 270 and 360 days, respectively. Tissue samples from *E. multilocularis* lesions were taken and processed for histopathological examination and immunostaining. In addition, liver tissue samples were taken 1) close to the parasitic lesions, i.e. 1–2 mm from the macroscopic changes due to the metacestode/granuloma lesion, thus avoiding liver contamination with infiltrating immune cells and parasitic tissue, and 2) distant from the parasitic lesion, in another lobe of the liver, in *E*. *multilocularis* infected mice; in control mice, samples were taken in the injected lobe and in a non-injected lobe of the liver.

#### Patients

In humans, the diagnosis of *E. multilocularis* infection (AE) was based on positive serology with ELISA using crude *E. multilocularis* antigen, Antigen B, Em2 and Em18 (Xinjiang Bei Si Ming, Urumqi, China) and characteristic liver lesions observed on ultrasound- and CT scans. All diagnoses were confirmed by histological examination of the lesions [35]; tissue samples taken for diagnosis were also used for immunostaining. In addition, to measure proteins in the liver using Western Blot and mRNA using real time RT-PCR, paired liver specimens (0.5 cm3 each) were obtained at surgery by an experienced surgeon from 16 patients with AE at the First Affiliated Hospital of Xinjiang Medical University, Urumqi, China. From each patient, one specimen was taken close to the parasitic lesions (0.5 cm from the macroscopic changes due to the metacestode/granuloma lesion), and one was taken in the liver distant from the lesions (the non-diseased lobe of the liver whenever possible, or at least at 10 cm from the lesion), according to a previously described procedure [35].

#### Processing of tissue samples

Liver samples were separated into two parts and either deep-frozen in liquid nitrogen for RNA isolation or formalin-fixed for histopathological examination. For histological and immunohistochemical studies, the liver samples were fixed in 4% paraformaldehyde in neutral buffered formalin for a minimum of 24 h, embedded in paraffin, and cut into 4 µm serial sections. Paraffin-embedded liver tissue samples of experimental mice and AE patients were stained by Hematoxylin and Eosin (H&E) and Masson’s trichrome for pathological observations.

### Immunohistochemistry Analysis

Immunohistochemistry was performed on formalin-fixed, paraffin-embedded tissue. Briefly, 4 µm tissue sections were de-paraffinized in xylene and rehydrated in gradual dilutions of ethanol. Endogenous peroxidase was blocked with 3% hydrogen peroxide. To increase staining, sections were pretreated by microwave heating for 15 min in antigen unmasking solution (pH 6.8, 0.1 M citrate buffer, Zhongshan Jinqiao Biology Corporation, Beijing). To block non-specific background, the sections were incubated with non-immune goat serum for 30 min. Sections were then incubated overnight at 4^o^C with the primary antibody diluted in pH 7.3 phosphate-buffered saline (PBS) (α-SMA 1∶200, Collagen 1 (COL 1) 1∶200, Collagen 3 (COL3) 1∶200, CD4 1∶100, CD8 1∶100, TGF-*β*1 1∶100, TGF-*β* receptor I (TGF-*β* RI) 1∶200, TGF-*β* receptor II (TGF-*β* RII) 1∶200, pSmad2/3 1∶200, Smad4 1∶200, and Smad7 1∶100) (Santa Cruz Corporation, CA, USA). After 3 washes in PBS, the sections were subsequently incubated with horseradish peroxidase conjugated host-specific secondary antibodies and 3, 3′-diaminobenzidine was used as chromogen. Sections were counterstained with hematoxylin for 5 min, dehydrated, and covered with slips. For all samples, negative controls consisted of substitution of the isotype-matched primary antibody with PBS.

### Western Blot Analysis

Western Blot analysis of cell lysates was performed by SDS-PAGE using NuPAGE (Invitrogen, California, USA) followed by transfer to nitrocellulose membrane (Invitrogen, California, USA). Ponceau S (Sigma, Missouri, USA) staining was used to ensure equal protein loading and electrophoretic transfer. Using the appropriate antibodies, TGF-*β*1, TGF-*β* RI, and RII, pSmad2/3, Smad4 and Smad7 (Cell Signaling Technology, Massachusetts, USA) and GAPDH (Santa Cruz Biotechnology, California, USA) were detected with WesternBreeze Kit (Invitrogen, California, USA). The expression levels of respective proteins (in “relative units”) in the liver of control mice and *E. multilocularis* infected mice, as well as in the liver of AE patients, were quantified using Quantity One software (Bio-Rad, Hercules, USA).

### Quantitative Real-time RT- PCR Analysis

After removing contaminated DNA from the isolated RNA using DNaseI (Fermentas, Vilnius, Lithuania), 1 µg of total RNA was reverse transcribed into cDNA in 20 mL reaction mixtures containing 200 U of Moloney murine leukemia virus reverse transcriptase (MMLV, Promega, Madison, USA); 100 ng per reaction of oligo (dT) primers; and 0.5 mM each of dNTPs, dATP, dCTP, dGTP, and dTTP. The reaction mixture was then incubated at 42°C for 1 hour and at 95°C for 5 min to deactivate the reverse transcriptase.

The real time RT-PCR was run in a thermocycler (iQ5 Bio-Rad, Hercules, CA, USA) with the SYBR Green PCR premix (TaKaRa, Dalian, China) following the manufacturer’s instructions. Thermocycling was performed in a final volume of 20 µL containing 2 µL cDNA and 10 pM of each primer (Table 4 and 5). To normalize for gene expression, mRNA expression of the housekeeping gene GAPDH was measured. For every sample, both the housekeeping and the target genes were amplified in triplicate using the following cycle scheme: after initial denaturation of the samples at 95°C for 1 min, 40 cycles of 95°C for 5 s and 60°C (or other) for 30 s were performed. Fluorescence was measured in every cycle, and a melting curve was analyzed after the PCR by increasing the temperature from 55 to 95°C (0.5°C increments). A defined single peak was obtained for all amplicons, confirming the specificity of the amplification.

**Table 4:** Primers and cycling parameters of real time RT-PCR detection of TGF-*β*1 signaling pathway (mouse).

Gene	Genbank Accession	Primer Sequences	Annealing Temperature	Expected Size
TGF-β1	NM_011577	F:5′-GTGTGGAGCAACATGTGGAACTCTA-3′ R:5′-TTGGTTCAGCCACTGCCGTA-3′	52.1°C	143 bp
TGF-β RI	NM_009370.2	F: 5′-TGCAATCAGGACCACTGCAATAA-3′ R: 5′-GTGCAATGCAGACGAAGCAGA-3′	60.0°C	133 bp
TGF-β RII	NM_009371.2	F: 5′-AAATTCCCAGCTTCTGGCTCAAC-3′ R: 5′-TGTGCTGTGAGACGGGCTTC-3′	60.0°C	100 bp
Smad2	NM_010754	F: 5′-AACCCGAATGTGCACCATAAGAA-3′ R: 5′-GCGAGTCTTTGATGGGTTTACGA-3′	60.0°C	198 bp
Smad3	NM_016769	F: 5′-GTCAACAAGTGGTGGCGTGTG-3′ R: 5′-GCAGCAAAGGCTTCTGGGATAA-3′	60.0°C	150 bp
Smad4	NM_008540	F: 5′-TGACGCCCTAACCATTTCCAG-3′ R: 5′-CTGCTAAGAGCAAGGCAGCAAA-3′	60.0°C	136 bp
Smad7	NM_001042660	F: 5′-AGAGGCTGTGTTGCTGTGAATC-3′ R: 5′-CCATTGGGTATCTGGAGTAAGGA-3′	60.0°C	126 bp
β-actin	NM_007393	F: 5′-AACTCCATCATGAAGTGTGA-3′ R: 5′-ACTCCTGCTTGCTGATCCAC-3′	56.0°C	248 bp

**Table 5:** Primers and cycling parameters of real time RT-PCR detection of TGF-*β*1 signaling pathway (Human).

Gene	Genbank Accession	Primer Sequences	Annealing Temperature	Expected Size
TGF′-β1	A23751.1	F:5′-ACACCAACTATTGCTTCAG-3′ R:5′-TGTCCAGGCTCCAAATG-3′	55.7°C	159 bp
TGF-β RI	NM_001130916	F: 5′-AATTCCTCGAGATAGGCCGT-3′ R: 5′-TGCGGTTGTGGCAGATATAG-3′	60.0°C	244 bp
TGF-β RII	NM_003242.5	F: 5′-AACAACATCAACCACAACACAG-3′ R: 5′-CCGTCTTCCGCTCCTCAG-3′	56.2°C	250 bp
Smad2	NM_005901.4	F: 5′-GTCTCTTGATGGTCGTCTC-3′ R: 5′-GGCGGAAGTTCTGTTAGG-3′	53.3°C	249 bp
Smad3	NM_001145104.1	F: 5′-GTGCTCCATCTCCTACTAC-3′ R: 5′-CCTCTTCCGATGTGTCTC-3′	56.5°C	183 bp
Smad4	BC002379.2	F: 5′-CAGGACAGCAGCAGAATG-3′ R: 5′-CAATACTCAGGAGCAGGATG-3′	55.6°C	232 bp
Smad7	NM_001190823.1	F: 5′-TTTGTGTATTTATTTCTTTCTCTC-3′ R: 5′-CACTCTCGTCTTCTCCTC-3′	54.5°C	194 bp
β-actin	NM_002046.3	F: 5′-GCACCGTCAAGGCTGAGAAC-3′ R: 5′-TGGTGAAGACGCCAGTGGA-3′	50.8°C	138 bp

### Expression of the Data and Statistical Analysis

Immunostaining of *α*-SMA, Collagen I, Collagen III, CD4 and CD8 was semi-quantified by calculating “expression scores” that consider both staining intensity and the percentage of cells stained at a specific range of intensities. A score of zero indicated the percentage of positive cells <5%, 1+ = 5–25%, 2+ = 25–50%, 3+ = 50–75%, 4+>75%. The staining intensity of each specimen was judged relative to the intensity of a control slide including an adjacent section stained with an irrelevant negative control antibody that was matched by species and isotype to the specimen. Staining of the section labeled with the negative reagent control was considered as background. A score of zero indicated no staining relative to background, 1+ = weak staining, 2+ = moderate staining, and 3+ = strong staining. According to standard pathology practice, staining intensity was reported at the highest level of intensity observed in all tissue elements, except the distinctive tissue element for which an expanded scoring scheme was reported. The “expression scores” were calculated by multiplying the percent of positive cells (0–4) and the staining intensity scores (0–3). For example: for a specimen with 30% of positive cells (3+), and a moderate staining intensity (2+), the “expression score” was 3×2 = 6. Three pathologists read the sections and established the scores, and they were blinded to each other results. Immunostaining of TGF-*β*1/Smads was quantified by calculating “positive cells”. Cells with a positive immunostaining were counted in five random visual fields of 0.95 square mm each, at initial magnification: ×20, for each sample, and the result was expressed as the percent of positive cells to the total number of cells counted.

All the data were analyzed by SPSS 17.0. The results were presented as means ± SD. One-way ANOVA and Student’s *t*-test were used to compare the differences between groups, and Spearman’s rho was used to analyze the correlation coefficient. *P*<0.05 was considered to indicate statistical significance.
